# The voice of the self: a typology of general practitioners’ emotional responses to situational and contextual stressors

**DOI:** 10.1080/02813432.2022.2097616

**Published:** 2022-07-13

**Authors:** Linus Johnsson, Lena Nordgren

**Affiliations:** a Department of Public Health and Caring Sciences, Uppsala University, Uppsala, Sweden; b Centre for Clinical Research Sörmland/Uppsala University, Eskilstuna, Sweden

**Keywords:** General practitioners, family practice, occupational stress, physician-patient relations, health policy, grounded theory, Sweden

## Abstract

**Objective:**

To develop a comprehensive typology of emotional reactions associated with stress among general practitioners (GPs), grounded in their own experiences.

**Design:**

Data was generated using observations and unstructured interviews, using Straussian grounded theory as the overarching methodology. The typology was built using multidimensional property supplementation.

**Setting:**

Eleven health care centres in urban and rural communities in four Swedish regions.

**Subjects:**

Sixteen GPs and GP residents.

**Main outcome measures:**

Characteristics of GPs’ emotional reactions in everyday work situations.

**Results:**

Accounts of negative emotions connected to stress revealed four principal personal needs of the GP: trust, efficacy, understanding, and knowledge. Simultaneous threats to more than one of these needs invariably increased the level of tension. From these more complex accounts, six second-order needs could be identified: integrity, judgment, pursuit, authority, autonomy, and competence. The most extreme encounters, in which all four principal needs were threatened, were characterised by the experience of being reduced into an assistant.

**Conclusion:**

The considerable resilience of GPs may belie some of the pressures that they are facing while being far from a fail-safe defence against being diverted from purposeful and morally responsible action. Our typology distinguishes between different forms of stress that may affect how GPs carry out their work, and connects to the vast literature on GP wellness. The results of this study could be used to develop tools for self-reflection with the aim of countering the effects of stress, and are potentially relevant to future research into its causes and consequences.

**Key points**
What is known
•Stress among GPs may have severe consequences for themselves and their patients, and levels of stress appear to be increasing.
What this article adds
•Stressful situations threaten at least one of four principal needs of the GP: trust, efficacy, understanding, and knowledge.
•More complex threats increase the level of tension and bring out second-order needs: integrity, judgment, pursuit, authority, autonomy, and competence.
•The wealth of literature on GP stress can be clearly understood through the lens of our four-dimensional typology.

## Introduction

Among the 11 countries participating in the 2019 International Health Policy Survey, Swedish general practitioners (GPs) reported the highest levels of stress despite seeing fewer patients per working hour than their colleagues abroad, and ostensibly enjoying the benefits of multi-professional collaboration and cutting edge IT support systems [1]. Swedish GPs report a worse psychosocial work environment than other health care professionals, possibly because they spend much time on administrative tasks [2]. The situation seems comparable to—and possibly worse than—the one in Norway, where the proportion of GPs reporting risky levels of work stress has increased from 10% to 40% in nine years [3], and the UK, where GPs describe their workload as unsustainable [4].

Regardless of how stress among GPs is conceptualised, the evidence on its harmful consequences is overwhelming. Job stress has been found to hamper practice performance [5], holistic care [6], and attention to less urgent chronic conditions [7]. High demand and low work control are associated with impaired general health and lower well-being [8] as well as adverse physician reactions such as burnout and intent to leave the profession [9]. Stressed physicians are less empathetic [10,11] and more prone to error, while those who are burned out are more likely to engage in unprofessional behaviours and hold less altruistic views [11]. Stress among GPs may, due to their compensating strategies, not always be overt; they tend, for instance, to take shorter breaks than their co-workers [2] or skip breaks entirely to cope with a heavy workload [12].

Some explanations for the increase in work-related stress among GPs can be found in societal changes and health care reforms of late. While reforms that uphold important values such as access or continuity may not affect stress or reduce job satisfaction [13], more recent trends toward unilateral transfer of tasks from secondary to primary care may increase the GP’s workload significantly [4,12]. In the UK, requirements to meet targets further contribute to GP workload [4], whereas in Sweden, guidelines are rarely associated with reimbursement schemes and tend to be perceived as largely benign [14].

In encounters with patients, stress may be caused by uncertainty and fear of making mistakes, which can hardly be avoided [15] yet is highly related to depression among GPs [16], or the time pressure that ensues when several patients compete for attention or when a single patient presents with multiple issues [7]. Although physicians are trained to make extensive use of themselves in the practice of their art [17], experiences from Balint sessions suggest that they may also struggle with their own subjectivity in relation to the other; they might, for instance, not always be perfectly clear on the position that they are speaking from [18]. GPs regularly face hostility and even violence when denying patient requests [19] or as part of a pattern of perpetually boundary-transgressing behaviour [20]. Even less aggressive patients can, through inappropriate requests, induce cognitive dissonance [21], feelings of compromised autonomy [22], or destructive collusion [23].

Stress among GPs is clearly multifaceted and complex, not only with regard to its causes, but also in terms of human experience. What appears to be lacking is a comprehensive account of the emotional reactions that shape GPs’ experiences and affect their choices. In the present article we shall attempt to address this lack by developing the concept of the *voice of the self*, which is part of our emerging theory of quality from the perspective of GPs [24], into a typology that encompasses and distinguishes between various types of strain. If this is feasible, we might be able to better understand how aspects of individual encounters and features of the work environment interact to affect the work of GPs.

## Methods

We used grounded theory according to Corbin and Strauss [25] as the overarching methodology; hence, data generation, analysis, and theoretical integration were carried out in parallel. In order to increase our chances of developing a novel conceptual apparatus grounded in data, most of the literature review, especially that of more theoretically inclined research, was delayed until after initial analysis.

The first author (LJ), himself a GP as well as a bioethicist, endeavoured to maintain an insider perspective, empathising with the informants to enhance theoretical sensitivity. As this might complicate distinguishing the informants’ voices from that of the researcher, the second author (LN), with a background in nursing and extensive experience of conducting qualitative research but limited first-hand experience of primary care, maintained openness by continuously challenging the interpretations of LJ and suggesting alternatives.

### Participants

The intended population comprised general practitioners in Sweden, including GP residents (’ST-läkare’). Although the latter have, by definition, not yet completed the specialist medical training required to become a GP in Sweden, they work in the same context as their seniors and can, at least to some extent, be expected to share their commitments and ethos. Furthermore, they might contribute with unique data due to their limited experience. For simplicity, both GP residents and GPs proper are referred to as ‘GPs’ in this article.

We recruited sixteen participants (eight women, eight men; eleven GPs proper, five GP residents), first by convenience and later guided by theoretical sampling. Potential participants were identified through personal knowledge and informal networks of GPs, and contacted by e-mail. The participants worked in 11 public and private healthcare centres, each employing from three to around 30 GPs, in urban areas, townships and remote rural areas, in four counties. We thus strived to strike a balance between contextual diversity and a partial overlap that could be used to draw conclusions about the impact of contextual factors.

### Data generation

Data was generated in 2015–2017 through observations and individual interviews carried out by LJ. As dictated by the method of constant comparisons, new data were continuously compared to previously sampled data to uncover similarities and differences.

We observed the participants for one-half to a full working day. Field notes were taken continuously and fleshed out later. The interviews were unstructured, audiotaped, lasted for 30–60 min, and focused on the participants’ experiences of their interactions during the day. We sought, as far as possible, descriptions of concrete examples (or ‘cases’) and therefore guided the participants towards topics in which we had a research interest. Beyond this, we did not restrict in any way what could be discussed. LN familiarised herself with the data by transcribing the interviews verbatim.

In grounded theory research, the researcher does not sample individuals as such, but rather ’events’ or ’cases’ that bring out the main concern of the participants. Sampling continued until we reached theoretical saturation of the main concepts of the process. Field notes and transcripts were split and remerged into 471 events, each of which could be read as a narrative about an encounter or a part thereof.

### Analysis and theory building

In keeping with Straussian grounded theory, analysis progressed through open, axial, and selective coding. We continuously recorded in memos our reactions to what we had heard or observed as well as our thoughts on the research process. These memos aided reflective understanding of our preconceptions, in particular those of the first author.

During open coding, we assigned one or several codes to each event to capture our initial understanding. Both researchers were intimately engaged with the data and strived for consensus regarding interpretations. Axial coding saw abstract yet meaningful codes, such as the *voice of the self*, evolve into categories that subsumed a large number of more concrete codes. Such categories were either corroborated or rejected through constant comparisons with subsequently generated data. To account for the heterogeneity of data within each category, we developed properties that captured practically significant differences between cases.

Lastly, we coded selectively by organising the categories around a core category—the GP’s *choice of maxim of action* [24]—that had intimate ties to all other categories. The core category of our theory describes the divergent norms that exert pressure on the GP and how acting on some of those may require others to be sacrificed. Within the first sub-study, the practical significance of the *voice of the self* became evident in how it easily subsumed experiences connected to strain as well as raised questions regarding the connection between such strain and subsequent action. Because it was a complex concept in need of a comprehensive typology to account for practically relevant variations in its manifestations, LJ analysed it further using multidimensional property supplementation (MPS) [26]. This method allows the researcher to easily switch back and forth between different aspects of the concept by varying the set of properties that are currently being considered. One criterion for selecting properties was that they must carry implications for subsequent action, as captured by the core category. We sought, in other words, a conceptual model that would distinguish between different experiences of strain only insofar as those differences could, at least hypothetically, affect the GP’s decision making.

Because of the emerging theory’s relatively high level of abstraction, traditional ‘member checking’ of interpretations of specific events was not a major methodological concern. Ascertaining relevance and clarity in the eyes of the target population was, in contrast, crucial; to this end, we presented and received feedback on early versions of the typology at international and national conferences (in 2019 at the Nordic Congress of General Practice, Aalborg, Denmark, and in 2021 at Svensk allmänmedicinsk kongress, Åre, Sweden), during research seminars at Uppsala University, and through informal collegial discussions.

### Ethical considerations

Oral and written informed consent was obtained from participating GPs. The information included details about the purpose of the study, its methodology, and the types of data that would be generated.

Our presence in the consultation room could be expected to change the dynamics between patient and doctor to some extent. Patients were protected from potential harm by asking their permission before entering the consultation room, and by encouraging the participants to exercise their right and responsibility to veto the presence of the researcher if deemed problematic. Even so, there is no telling whether some patients would have preferred to see their GP in private.

Although we did take field notes on the observed patient–doctor encounters, we took care to avoid recording any direct or indirect patient identifiers. Because we were interested mainly in the GP’s reactions, ethical deliberation, and actions, we did not record details about the patients’ health beyond simple labels to aid later recollection.

The study was exempted from review by the regional research ethics committee in Uppsala (Dnr 2015/030).

## Results

The encounters observed or reported to us revealed, besides many positive experiences associated with human interaction, also various types of negative emotions—indignation, worry, frustration, helplessness—in response to professional, contextual and situational demands. Some informants described how their basic human reactions had, during past or present encounters, affected their behaviour in ways that they took no pride in and sometimes regretted. At other times, paradoxically brief comments revealed profound resignation in the face of loss of professional authority or the deterioration of their work environment. A shared feature of these experiences is that they point out threats to the self that are deeply personal in the sense of being inescapable through detachment.

### The ideal situation

*You want to communicate to this person a feeling that all is well. They should be in better health when they leave than when they arrived, shouldn’t they, with all their worries and ruminations. (Senior GP, male)*

The *voice of the self* is best understood by comparing stressful situations against a baseline of sorts: the (from the GP’s point of view) ideal, uncomplicated, *unthreatening* consultation. We observed, indeed, some medically clear-cut cases, where the GP was trusted, knowledgeable, and able to make a difference through straighforward action. Other ideal consultations revolved around accumulating or using idiographic knowledge, by virtue of which the GP might be better situated to ‘relieve often and comfort always’ than their hospital-based colleagues despite, perhaps, inferior medical expertise. Even emotionally laden encounters were not necessarily stressful. One senior GP reflected on how relatively harmless conditions might cause dramatic symptoms that must be expertly handled lest the patient’s troubles be compounded by anxiety, a task which, given the right set of communication skills, was relatively straightforward. In another memorable encounter, a senior GP engaged in open-hearted conversation with a seriously ill patient on the art of living with illness, a weighty topic that they nevertheless seemed to find deeply satisfying.

### Four principal personal needs

**Figure 1.**
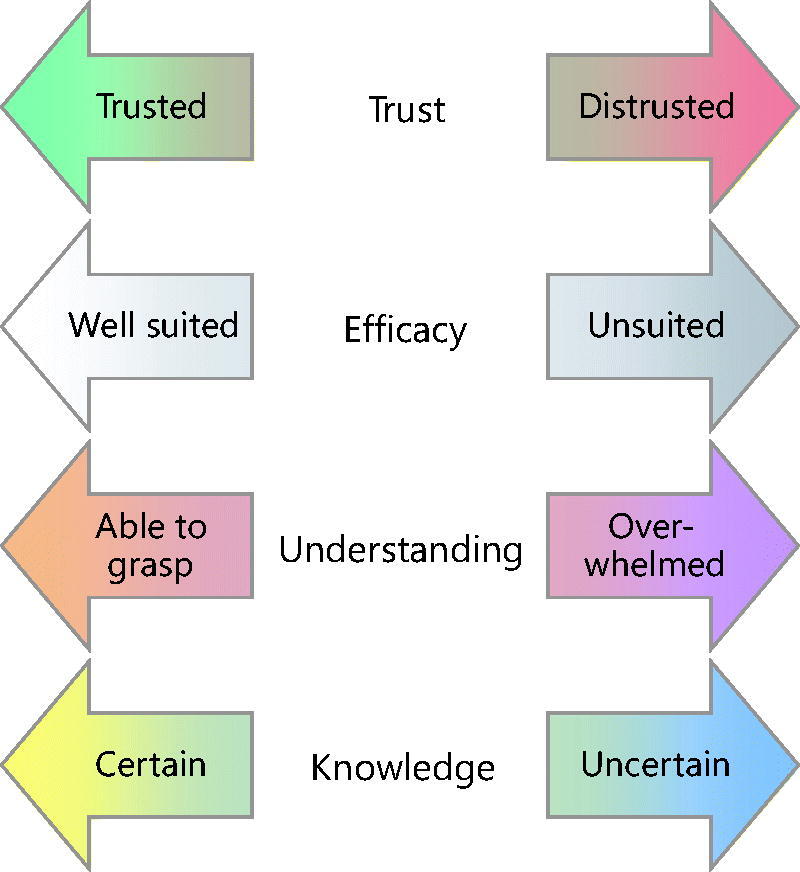
The GP has four personal needs that are ‘principal’ in the sense of being mutually independent: *trust* (being listened to, given the benefit of the doubt, and receiving recognition for their work), *efficacy* (being well suited to the task and in a position to make a difference), *understanding* (being able to grasp the essential features of the problem, including the values at stake), and *knowledge* (having general medical knowledge and specific facts about the situation).

The *voice of the self* accounts for the GP’s negative emotional responses by positioning them within a four-dimensional space of possibilities defined by four axes, each of which corresponds to a personal need: *trust*, *efficacy*, *understanding*, and *knowledge* (see Figure 1). Our emerging theory predicts that threats to any of these four needs will trigger negative emotions that affect the GP’s moral decisions.

In what follows, we describe situations that involve threats to the four principal needs individually, before moving on to more complex cases.

### Trust

*I don’t believe in being paid to ask people about their smoking habits. It generates the wrong motives, we become prone to do the wrong things. (Senior GP, male)*

Although a trusting relationship was the norm, there were also instances of *distrust*, mostly experienced in one of three different forms. First, patients might present with set agendas—demanding, for instance, some specific investigative procedure, treatment, or sick-leave certificate—while regarding the GP merely as an obstacle to be surmounted. Second, hospital-based colleagues sometimes seemed to distrust the GP’s judgment by giving low priority to their referrals or rejecting them outright. Third, the myriad of guidelines, policies, and checklists infringing on the GP’s work could be perceived as betraying an underestimation of their competence as well as of the complexity of real-life situations. The core experience of being distrusted, present in all three forms, can be captured theoretically as being unneccessarily told how to do one’s job.

### Efficacy

*That I find a little bit hard, when they are … complaining and wanting you to help, and you don’t have all that much to offer beyond listening. (Senior GP, male)*

When caring for patients with chronic debilitating conditions who, after many failed attempts at treatment, would continue pleading for help, GPs experienced an emotionally taxing lack of efficacy, or being *unsuited* to the task. Even in objectively more hopeful cases, GPs sometimes found themselves fighting an uphill battle against expectations to ‘fix’ conditions that would require interventions beyond their abilities, such as physiotherapy or psychotherapy. Unrealistic expectations could also originate from the system in the form of demands to effectively treat therapy-resistant issues such as smoking or obesity. What unites these examples is the experience of being charged with responsibility for problems against which one cannot effectively intervene.

### Understanding

*You have to leave the room and, like, supervise in parallel … it’s part of the deal, yet it is an interruption … and then you'll have to start over. (Junior GP, female)*

The GPs expressed a need to understand the nature of the problem before them, including the values at stake. An abundance of data squeezed into a tight time frame could easily become *overwhelming*. As an example, encounters with familiar patients tended to follow an established script, part of which had the patient present a long list of complaints. Although many patients recognised the need to prioritise (and even cooperated in the effort), an element of time pressure would often linger. A somewhat similar situation occured in the very different context of unscheduled sessions; as these consultations were expected to be straightforward, one or two complex ones might be enough to cause an irrecoverable delay. Lastly, GPs, in particular those who were supervising junior colleagues, regularly lost precious time and focus due to interruptions. As a whole, the experience of being overwhelmed occurs when the GP cannot grasp the situation within the allotted time frame.

### Knowledge

*You can’t investigate everything in every way possible … there aren’t enough resources for that, nor does it feel ethically justifiable to put your patients through a lot of unnecessary examinations. (GP resident, female)*

Uncertainty was common, and generally due to lack of useful information about the case at hand. Because of the probabilistic nature of diagnosing illness, the GP might experience uncertainty even when all facts were supposedly on the table, especially when unbalanced by deviations from the expected chain of events. Patients who described their symptoms in non-standard ways or failed to follow agreed-upon treatment regimens also caused difficulties. Junior doctors were generally more prone to second-guessing their decisions and faced, as a consequence, significant challenges in deciding on appropriate lines of inquiry and dealing with unexpected results. Being uncertain carries, in sum, a connotation of risk, but also a sense of responsibility and ability to mitigate it.

### Second-order needs of the self

**Figure 2.**
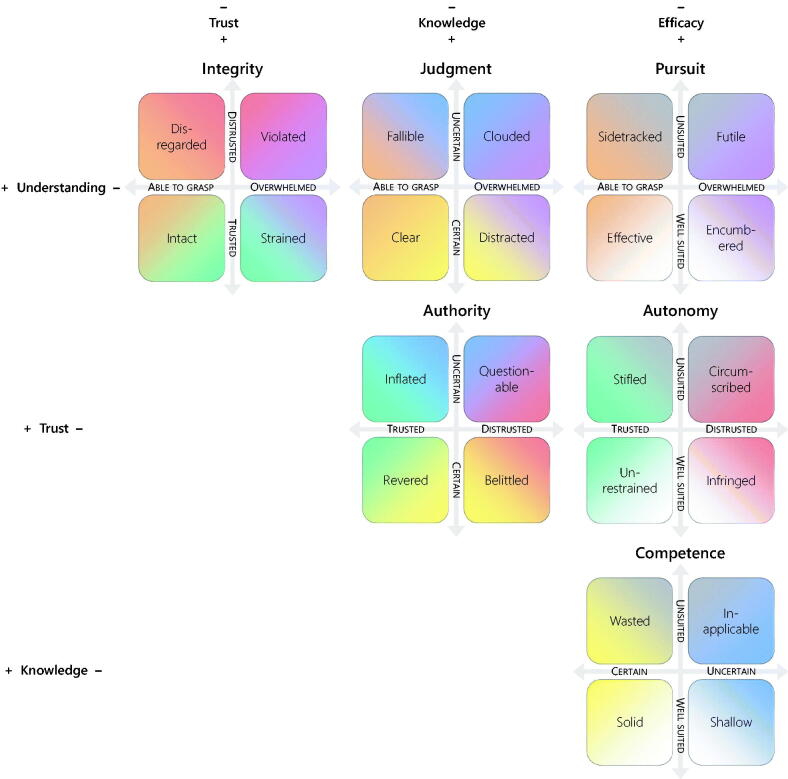
The four principal personal needs of the GP can be combined into two-dimensional projections, each of which corresponds to a ‘second-order’ need: *integrity* (maintaining certain boundaries against the outside world); *judgment* (making decisions informed by both facts and moral imperatives); *pursuit* (being able to effect valued change); *authority* (receiving recognition for one’s knowledge); *autonomy* (being free to set professional goals); and *competence* (being knowledgeable about those matters that one is well suited to handle). Because they share components (principal needs), second-order needs are highly interdependent.

Because the four principal personal needs and their corresponding threats are logically independent, it is possible to construct a four-dimensional model that considers variations along each axis simultaneously. Juxtaposing pairwise combinations of axes in a series of two-dimensional projections was useful not only methodologically as a stepping stone to the fully-fledged model, but also theoretically because these projections could be understood as second-order needs of the GP (see Figure 2).

Second-order needs can be most clearly understood by attending to the common traits of situations where both component needs are threatened, whereas the two remaining needs are unthreatened. In what follows, each second-order threat is explained, first by example in order to ground it in data, and thereafter through theoretical explication to demonstrate its theoretical relevance.

### Integrity

*Nobody can do their work, can they, if they’re constantly interrupted by trivialities. (Senior GP, male)*

The sanctity of the consultation, as it were, was occasionally violated by rude interruptions for trivial or nonurgent purposes, or by patients who were particularly insistent on bringing up, or unwilling to move on from, particular issues. On a larger scale, the GP’s integrity was perpetually challenged by hospital specialists presuming their assistance with housekeeping tasks, such as ordering tests or writing sick-leave certificates. More insidious was the tendency of some GPs to disrespect their own limits by normalising a state of constant hurry.

*Integrity* is about the boundaries between oneself and the outside world. It remains *intact* as long as the GP sufficiently understands the situation and enjoys trust from involved actors, as this allows them to stand apart from the problem while remaining in close enough contact to make learning possible. Integrity can become *strained* by an overwhelming amount of data or tasks, or *disregarded* by those who seek to use the GP for their specific agendas. An actual *violation* of integrity arises through unwelcome intrusion into temporal or physical personal space. When this happens, mounting an organised defence is difficult because the GP lacks both a coherent understanding of the problem and the social leeway to probe and question it.

### Judgment

*I tried to ask her, ‘What’s most important?’ Well, the neck pain. And then it turns out that the lung issue was the one that really mattered to her. And then again, most important in the end was perhaps getting a prescription. (GP resident, female)*

Patients, especially those who were previously unfamiliar to the GP and suffered from chronic conditions with unresolved issues, sometimes presented mystifying or ambiguous complaints, or carried agendas that clashed with the GP’s need for clarity. In such situations, trade-offs had to be made between resolving uncertainties and saving time. Younger doctors worried more about potentially overlooking crucial information, and were thus more likely to prolong the interview or conduct an extensive physical examination. GPs supervising junior doctors faced a similar quandary, but generally leaned in the other direction because checking all the facts themselves would be prohibitively time-consuming.

*Judgment* is about one’s ability to make decisions informed not only by facts but also by what is morally required. The ideal, *clear* judgment, implies being certain about facts and sufficiently understanding the problem. In the face of *fallible* judgment, the gaps in one’s knowledge are still identifiable and remediable, whereas *distracted* judgment can be recovered by using one’s general knowledge to sift through the facts. When there is both an abundance of data and a dearth of information that actually provides guidance, judgment becomes *clouded*, the hallmark of which is that the process of selecting the uncertainties that need to be addressed becomes complicated and error-prone.

### Pursuit

*When it comes to preventive work, which I suppose is key, really … it’s difficult to … well, you know, the patient often wants immediate relief. (GP resident, female)*

Some GPs appeared to have become everyone’s go-to person for medical advice. Although flattering, this tended to dilute their work and leave little time to address more pressing concerns. Analogously, individual encounters were occasionally flooded with insoluble or trivial problems that distracted from or obscured more promising or weighty ones. One senior GP, longing for more complex and challenging cases, spoke candidly about being bored with routine checkups for chronic conditions, issues that would be better handled by more eager junior physicians.

*Pursuit* denotes the GP’s value-driven effort to effect change. Ideally, it is *effective*, which can only be the case when they understand the problem and possess basic efficacy in handling it, but can become *encumbered* by the need to juggle information and tasks, or *sidetracked* when there are few meaningful decisions to be made, as when expectations are fixed. Lastly, the GP’s pursuit becomes truly *futile* when precious time is wasted on tasks that should be handled by others but which the GP cannot ignore if they wish to proceed with what truly matters.

### Authority

*I suppose he … expected me to take on the role of doctor House or something, who says, ’It could be this! We’ll investigate and then you can have this treatment and recover.’ (GP resident, male)*

Trained to always approach the patient with an open mind, GPs were occasionally rebuked by patients who expected them to have a clear pre-understanding of their problem, or who were disinclined to forgive any signs of uncertainty regarding the nature of their illness or possible effects of treatment. Others expected the truth to be revealed through blood tests or x-rays and were suspicious of the GP’s apparent reliance on history taking. Attempts to capture and quantify concerns were sometimes met with monosyllables or even open hostility. Patient access to medical records, a rather novel phenomenon at the time, made dealing with uncertainty harder for some GPs, who now had to think twice before recording their hypotheses.

Questions about *authority* arise in the intersection between the GP’s knowledge and public recognition of that knowledge. Ideally, their authority is *recognised* through adequate trust in their actual abilities. Authority can become *inflated* through overblown expectations, or conversely, *belittled* by hopes that are set too low, either ingenuously or ingeniously. The difficult situation of *questionable* authority occurs when the GP is the target of reasonable doubt. Given that displaying weakness in such conditions is difficult, the GP is at risk of being lured into pretense of omniscience rather than admitting ignorance.

### Autonomy

*About these treatment targets … They are touted as the state of the art … but nobody cares if people feel unwell because their blood pressure is way down. (Senior GP, male)*

GPs reacted with indignation against being encouraged to pursue system goals that they perceived as less important than the task at hand. Top-down initiatives to improve care, such as the administration of surveys on health behaviours, were conceived of as simplistic and unfounded. One senior GP spoke vehemently about being cast in the role of provider of services for purely bureaucratic or economic purposes. Novelties perceived to carry little medical value (such as general health checks) or merely situational benefits (such as video consultations) produced simliar, although less emphatic, reactions.

The GP’s *autonomy* is, on a minimal understanding, about their freedom to set professional goals and select tools for those purposes. The ideal from their point of view, *unrestrained* autonomy, requires efficacy with regard to the problem at hand and trust from other actors. Autonomy *stifled* by a lack of meaningful choices might still be recoverable thanks to a trusting environment, whereas autonomy *infringed* by a need to step carefully can be at least tolerable because what the GP is expected to do happens to be valuable, if only contingently. *Circumscribed* autonomy, in contrast, implies being diverted from the most worthy problems or the most promising solutions and steered instead towards tasks that are either pointless or could be better handled by others.

### Competence

*I recall telling her, ’You don’t need to worry about this, but it needs to be removed, and I don’t feel confident doing surgery on your neck so I’m sending you to ENT.’ (Junior GP, female)*

GPs were often at a knowledge disadvantage relative to specialist nurses educated about in-vogue treatments, or sometimes even patients after their latest web search. Such situations were problematic because the other party would tend to underestimate the value of the GP’s general knowledge and experience and, as a consequence, fail to be swayed even by well-founded theoretical arguments. The dynamics were slightly different in the context of rare conditions. Senior GPs were often quite content to refer such patients away, taking instead pride in their expertise in handling more common (yet complex) situations. Their juniors, in contrast, were more inclined to assimilate new knowledge and retain responsibility, but also struggled more to find their bearings, for while they might receive case-by-case advice from hospital-based colleagues, they could not always expect to be taught more esoteric and generally applicable knowledge and strategies.

GPs continuosly measure themselves against an ideal *competence*, the idea of which is nebulous but at least partly shared. A *solid* competence requires knowledge with regard to matters where the GP is also efficacious. It is *shallow* (but feasibly improvable) when the subject matter is obscure to them yet within legitimate norms of expertise, and *wasted* when their skills in problem-setting or problem-solving are under-utilised. The GP’s competence becomes *inapplicable* when they step outside the framework by which they normally address problems. In such situations, problem-setting is nigh impossible due to insufficient knowledge, and any effort to learn will be hampered by lack of direction.

## Complex threats to the self

**Figure 3.**
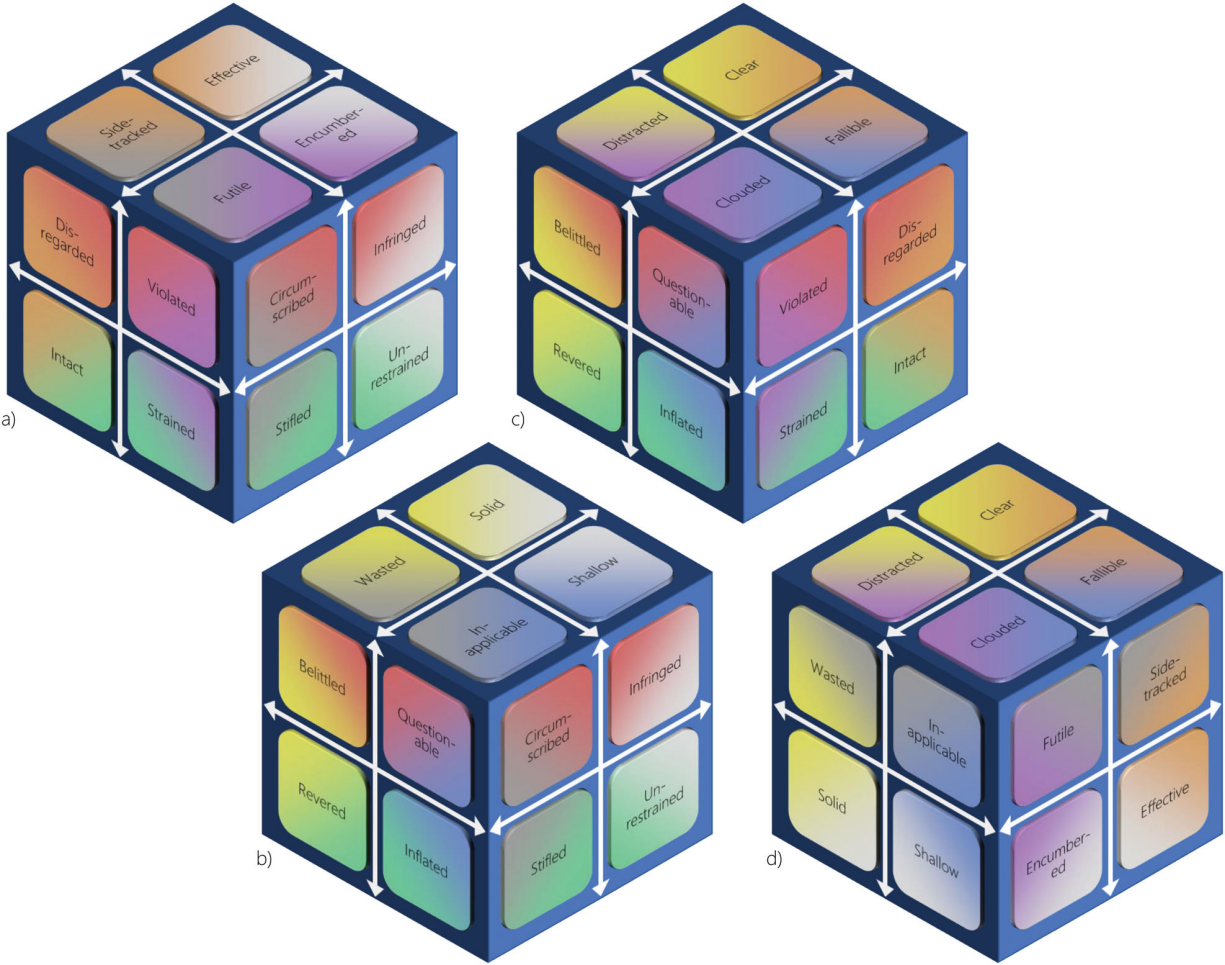
The four possible third-order threats to the self can be understood as being a) *deprofessionalised* (violated integrity, futile pursuit, and circumscribed autonomy), b) *deprived of identity* (questionable authority, circumscribed autonomy, and inapplicable competence), c) *exploited* (violated integrity, clouded judgment, and questionable authority), and d) *bogged down* (clouded judgment, futile pursuit, and inapplicable competence). The fourth-order threat of *being reduced into an assistant* implies the simultaneous presence of all third-order threats.

As might be becoming apparent, the robustness of the typology depends on a clear understanding of each of the six two-dimensional projections. To complete the picture, we shall briefly describe more complex situations that can be construed as third- or fourth-order threats to the self (see Figure 3).

### Deprofessionalised

*I have experience of managers … well, they make decisions and have opinions but they aren’t the ones who are affected, are they? If the computer acts up, they might say, ’It’s not a big deal, is it, you can fix that later, can’t you?’ No, because you won’t get your job done. Things have to work. (Senior GP, male)*

Managing without assistance, struggling with barely functioning equipment, cleaning up others’ messes, or engaging in purely administrative tasks were activities that were perceived not only as inefficient, but also as undignified. Less personal, but equally insulting, were the tendencies of other parties to unilaterally set deadlines with which the GP was expected to comply. Some GPs experienced losing power over their schedule, for instance by being expected to work over lunch breaks for little gain, as particularly distressing. What unites these examples is a form of deprofessionalisation, where the GP not only loses power over their time and labour, but is also used for purposes oblique to their mission.

### Deprived of identity

*When they started referring out, like, ’Checkup post MI,’ we had a discussion about that … that is, what is primary care, and what is secondary care. (Junior GP, female)*

In boundary negotiations, GPs were constantly under pressure to expand their responsibilities. While guidelines might be helpful in areas where they felt their competence lacking, there were also concerns regarding the attitudes underpinning them. Some GPs remembered occasions when hospital-based colleagues had, with precious little understanding of the context, expressed strong opinions on how consultations should be organised in order to improve care for their pet disease. As the GP has been forced to step outside of—or is at least balancing on the fringes of—their field of competence, such forms of critique cannot always be easily shrugged off, but cut instead to the core of their identity.

### Exploited

*I don’t know what he had expected, whether he thought that I should have filed the certificate much earlier … It sounded like we, well, it felt almost like, ’Why didn’t you do this earlier?’ (Senior GP, female)*

Patients with certain agendas—most notably, those who were fishing for addictive drugs or sick leave, or vying for the GP’s support in conflicts—tended to induce feelings of being deceived or manipulated. Similar reactions ensued when the GP, already working on a tight schedule, was assigned a novel and complex case without any option to refuse or redirect it. GP’s could not, for instance, reject referrals from hospital specialists even when lacking vital information, because this would amount to abandoning the patient. Crucial to the experience of being exploited is the feeling that one is being intentionally, or at least through indifference, intruded upon, yet kept in the dark as to the nature of the problem.

### Bogged down

*If there is a need of sick leave, you end up being involved … and you will keep passing the ball betwen, well, everyone in the team … (GP resident, male)*

When accosted with questions to which no medically sound answers could be formulated, GPs often sought to merely survive the encounter rather than actually contributing. Seeking assistance from other professionals was not always an option; when strained to the degree that they failed to see things clearly, the GP’s troubles might even be compounded, at least in the short term. Furthermore, the GP would often be stuck indefinitely with the responsibility for writing sick-leave certificates and prescribing medicines. These cases exemplify how the GP can become bogged down with problems that they can neither solve nor fully evade.

### Reduced into an assistant

*Quite frequently when referring a case to the hospital, even though the agreement says it belongs there, they respond, ’Order this and that examination, and that one,’ even though those are clearly specialist-level examinations. And then … you are supposed to send the results to the hospital specialist anyway. So you are really acting their secretary, aren’t you. (Senior GP, male)*

To the GPs that made mention of it, the trend over the past few decades of relocating care from hospitals to primary care was a mixed bag at best. Although assuming responsibility for patients traditionally managed by internists or psychiatrists had certainly made their work more interesting, they also experienced diminishing support from their hospital-based colleagues. Because the flow of referrals between GPs and their colleagues had now been partly reversed, the latter had gained a tool for dictating the GP’s actions through directives, masked as ‘suggestions,’ which could not be ignored lest the GP risk being denied further assistance. Some clinics even appeared to systematically dismiss referrals until the GP had carried out certain investigative procedures the results of which they would not know how to interpret.

Beneath the thin veneer of necessary organisational change one finds clear signs of power play. From this perspective, what goes on is an attempt to reframe the role of the GP by reducing them into an assistant who is not expected to provide any professional input of their own. Besides being inherently repulsive to them, this process systematically drains them of the resources that they would need to effectively challenge the state of matters.

## Discussion

In this article, we theorise the GP’s personal needs for trust, efficacy, understanding, and knowledge as a four-dimensional concept, and connect it to the vast literature on GP stress. While situations where a single need is threatened may be as ubiquitous as they are uncomfortable, the presence of multiple threats invariably increases the level of tension. From the four principal needs emerge six second-order needs—integrity, judgment, pursuit, authority, autonomy, and competence—that together shed light on the nature of the GP’s personal investment in their profession, as well as on that of their vulnerability to nefarious influence.

## Strengths and limitations

This study has two main strengths. First, as GPs are known to be highly resilient and adaptable in the face of stressors [27] and endeavour to keep low morale as a private experience rather than allowing it to dictate their behaviour [28], it was important to uncover emotional responses that might otherwise go unnoticed. The observer/interviewer (LJ), himself a GP and thus sensitised to the context, recognised the potential for emotional reactions (particularly negative ones) in many situations where overt emotions were lacking. Once the informants had developed trust in the researcher, they could afford letting off some steam and more easily discuss their reactions—even those that were initially ambiguous or obfuscated—without any fear of being misrepresented.

Second, as events indicating instances of the *voice of the self* were ubiquitous, diverse, and often complex, multidimensional property supplementation [26] turned out to be a method well suited to analyse them. To be precise, the potential vagueness of the concept could be transcended by combining its most basic building blocks—the four principal responses which, due to their high level of abstraction, might be hard to pin down reliably in real-life examples—into subspaces that were more evocative, yet more homogeneous than their parent concept.

Our study also has certain limitations. First, because it theorises patterns across GPs’ activities on a relatively high level of abstraction, it may provide little detail on the determinants of specific issues that GPs face in relation to some problem area of interest. Second, although we are fairly confident in the validity of the *voice of the self* in the sense that it tells us much that is true and important, one could be concerned about its reliability. The informants were not perfectly clear about their reactions at all times, and interpretation and coding were therefore highly researcher-dependent. This is, on the other hand, merely a special case of the observer-dependency that is already an intrinsic aspect of qualitative research. Its most likely consequence is a dilution of causal patterns, and this is not a major concern for the purposes of this article.

## Findings in relation to previous research

The four principal personal needs that constitute the *voice of the self* resonate well with previous research. *Trust* is immediately and obviously challenged by demanding patients [22]. *Efficacy*, understood as having opportunities to use one’s abilities, has been identified as an important source of satisfaction [29]. *Understanding* implies having adequate time relative to the complexity of the issue, which has been found to predict higher job satisfaction [30] as well as less stress and better patient enablement [31]. Lastly, *knowledge* is challenged by uncertainty, which predicts physician stress and burnout [32], plausibly because it instils a sense of vulnerability [33]. Interestingly, the revised four-factor version of the seminal demand–control–support model of job stress [34] makes no mention of uncertainty, perhaps because it might not be as strong a determinant of stress in most other job contexts.

The observed increase in strain with the number of simultaneous threats aligns well with the known association between multiple stressors and burnout [35] and compassion fatigue [36], the determinants of which are echoed in the literature on challenges to physician wellness: emotionally-charged situations, excessive cognitive demands and workload, increasing patient-care demands, growing bureaucracy, and standardisation and loss of autonomy [37]. Understanding how various kinds of everyday emotional strain interact to affect GP behaviour and long-term efficacy requires, we would argue, that one identifies those stressful aspects that can be considered atomary while staying true to the original experience. In what follows, we shall attempt such a theoretical contribution by mapping experiences of GPs, as they are reported in other studies, to the two-dimensional projections of our concept. By focusing on this intermediate level of abstraction we hope to paint a vivid, yet disciplined, picture of stress among GPs.

### Integrity

General practice has long struggled with its self-image, having been defined over the years either circularly (that which GPs do) or reductively (what their hospital colleagues do not do) [38]. This conceptual vagueness has real-life ramifications. The literature is replete with examples of GPs’ job satisfaction being challenged by health care reforms that transfer tasks to them without concern for their professional dignity or morale [4,12] or even patient safety [39]. In time-pressurised and incentivised contexts, some GPs feel that they are becoming less patient-centred and more goal-directed [40], which could explain why they occasionally fail to clarify their patients’ concerns [41] or resort to questionable prescriptions in order to maintain the high patient turnover which is valued by management [21].

It has been argued that the GP, unless they find a way to strike a balance between involvement and detachment, risk abandoning the patient in their time of greatest need [38]. In this study, we understand integrity as maintaining certain boundaries between oneself and one’s environment, while staying close enough to allow learning. This resembles the ideal of ‘detached involvement’ where one is simultanously involved with the patient and free from self-centred attachments [42] and contrasts with the ‘unboundaried’ role described by British GPs in the face of the rising tide of paperwork, culture of target management, and increasing patient expectations [43], as well as being a far cry from the kind of collusion that engenders powerlessness [23].

### Judgment

In the era of evidence-based medicine, the importance of professional judgment, although clearly argued [44], is easily forgotten. That access to facts is not enough to secure judgment is obvious to any physician who has ever been unclear about the reason for the consultation [41]. Good clinical decisions require, besides scientific evidence, also ‘imagination and an appropriate degree of emotional engagement’ [45], proper engagment with ethical and existential agenda [46], and a theoretical base that allows facts and evidence to be interpreted in the light of a wider range of values and human experiences [47].

One potential source of job dissatisfaction is complexity [30]. A possible mechanism, predicted by our emerging theory, is that high complexity increases the risk of becoming uncertain and overwhelmed, thereby posing a significant challenge to judgment. As an example, teamwork and role substitution, their benefits notwithstanding, reduce the GP’s opportunities for getting to know the patient [48] and introduce intermediaries which complicate the acquisition of useful information [49], possibly entailing more complex and stressful visits [1] and increased physician workload [50]. According to our conceptual model, these situations challenge the GP’s judgment not because facts are scarce, but because they complicate the GP’s efforts to establish a point of reference around which facts can be organised.

### Pursuit

GPs are highly concerned with making their work effective—to actually ‘make a difference’ [51]. They tend to be dissatisfied with their administrative tasks, which they conceive of as external to their profession [52], and ambivalent towards increased access, which may increase the frequency of visits for self-limiting conditions [53]. When called upon often and inappropriately, GPs find it harder to muster empathy [10] and may respond with taking shortcuts [54].

External distractions aside, finding the time to deal with high-priority issues can be a challenge even in the relative safety of the consultation room, since most patients bring up multiple problems even on short visits [55] and occasionally insist on recounting their situation in agonising detail [41]. The apparent consensus that framing the clinical question is ‘the most complex intellectual exercise in clinical medicine’ [56] is arguably merely a special case of the realisation that professional work requires, above all else, transforming problematic situations into workable problems [57]. Indeed, it is hard to see how an effective pursuit of values—which, according to our typology, implies an understanding of the problem and efficacy in handling it—would at all be possible without reflective practice.

### Authority

The nature of GPs’ work requires that they be able to tolerate a fair bit of uncertainty [33]. Although both disclosing and hiding uncertainty can be ways of tolerating it [58], there are indications of reluctance towards the former [41]. It has been suggested that doctors who carry self-images that are highly sensitive to perceptions of success may be particularly tempted by the mental strategies of 'infallibility’ and ‘authority’ [15]. It is beyond question that authority can be used nefariously, for instance to shame the other into submission [59]; it can also have severe consequences such as dogmatic authoritarianism, proceduralism, dogged adherence to rituals of clinical practice, and a tendency to ignore contrary evidence [60].

Although authority is partly about knowledge imbalance, it is all too easy to overplay this aspect, while neglecting its connection to responsibility. No matter how enlightened and empowered patients become, situations will arguably remain where the decision needs to be made by the physician [38]—within, of course, the bounds of patient consent. GPs have described how they use their authority benevolently to effect positive change, be it in cooperation with the patient or in a tug of war [61]. There is nothing inherently threatening about patients being knowledgeable; to the contrary, it has been found that instances where physicians react negatively to threats to their authority are characterised not by their patients being well-informed, but by distrust [20,62]. Similarly, our findings suggest that what kind of authority GPs bring to bear in their interactions hinges not only on their knowledge about medical matters, but also on the trust that they receive from patients, coworkers, and policy makers.

### Autonomy

Professional autonomy, defined as ‘individuals’ ability to control the terms and content of their work’ [30], may be the most consistent predictor of changes in GP career satisfaction [32,63–66]. Because autonomy is crucial to intrinsic motivation, performance, and worker well-being [67] and facilitates the integration of originally extrinsic values and regulations [68], this is unsurprising, as is the observation that ‘highly professional physicians will try hard to do the right thing even when they are not being measured’ [11], although this may run counter to popular belief.

In the present study, we theorise the principal threat to autonomy as being charged with tasks that do not further valued ends. Examples from the literature include being pressurised by patients into prescribing against one’s ideals [22], instructed by hospital colleagues to carry out inappropriate tasks [4,39], or having one’s attention diverted from important issues by medical protocols [10]. Suspicious attitudes towards evidence-based medicine may be caused by fear of being controlled through performance assessment [69]—a warranted fear, it seems, given that GPs have reported disregarding patient preferences in order to ‘get the numbers right’ for the audit [70] as well as coming to resent not only the assessment process, but also those noncompliant patients that lower their scores [71]. Seeing such cases as instances of the known vulnerability of autonomy to rewards perceived as controlling [67] is not far-fetched.

### Competence

Job satisfaction among GPs is associated with self-perceived clinical competence [72] and several corollary experiences: being intellectually stimulated, granted continual professional development, allowed to master a wider range of procedures, and given opportunities to take responsibility [73]. The image thus evoked bears more than passing resemblance to the ‘flow context’ of high skill and high demands, well-established as a source of joy and self-esteem [74]. Indeed, several of the more experienced GPs in our study took particular pride in ‘their own expertise,’ namely to manage particularly complex cases. Lack of competence, in contrast, is experienced as a threat to the self [22]. Unsurprisingly, GPs may prefer—circumstances permitting—to eschew responsibilities that they perceive as a waste of time [75] and tasks that others can do better [76].

The more severe threat that we have labelled ‘inapplicable’ competence may sometimes be an inescapable part of the bargain, for instance in rural contexts where GPs are forced to manage rare cases without backup [77]. More disconcertingly, Swedish GPs collaborate and coordinate more than their colleagues abroad, yet experience themselves as less competent in handling chronic conditions [1]. It is reasonable to ask whether this lack of self-confidence might be connected to the known perils of task shifting, namely, becoming removed from the interactions where one excels [38] and spending instead more time carrying out trivial tasks or dabbling in in fields of competence defined by others [39].

## Meaning of the study

This study carries implications for general practice researchers, GPs, and policy makers. Researchers might find the theoretical concept worthwhile as a framework for future research into determinants and consequences of GP stress. To GPs, it might be transformed into a tool for self-reflection, in particular with regard to the nature of their subjectivity and the impact that fulfilment (or non-fulfilment) of their needs—which they might otherwise ignore in order to maintain their professional ideals—may have on their work. To policy makers, it shows some of the complexity of the GP’s role in primary care and highlights the vulnerability of professionalism to organisational change—the kind of professionalism that management might regard with suspicion, yet which is an integral, and often taken-for-granted, ingredient of meaningful work.

## Conclusion

A GP’s work is inherently stressful. Although GPs possess a considerable resilience that belies the pressures that they are facing, this is not a fail-safe defence against being moved away from purposeful and morally responsible action. We found four principal threats to personal needs: trust, efficacy, understanding, and knowledge, from which a complex typology can be derived. When considering the interactions between these principal threats to the self, six second-order needs emerge that are morally relevant and connect to the vast literature on general practice and GP wellness: integrity, judgment, pursuit, authority, autonomy, and competence. Our four-dimensional model strikes a balance between comprehensiveness and simplicity that makes it potentially useful as a tool for self-reflection among GPs with the aim of countering the effects of stress, as well as relevant to future research into its causes and consequences.
